# Exposure of Unwounded Plants to Chemical Cues Associated with Herbivores Leads to Exposure-Dependent Changes in Subsequent Herbivore Attack

**DOI:** 10.1371/journal.pone.0079900

**Published:** 2013-11-20

**Authors:** John L. Orrock

**Affiliations:** Department of Zoology, University of Wisconsin, Madison, Wisconsin, United States of America; BASF Cropdesign, Belgium

## Abstract

Although chemical predator cues often lead to changes in the anti-predator behavior of animal prey, it is not clear whether non-volatile herbivore kairomones (i.e. incidental chemical cues produced by herbivore movement or metabolism but not produced by an attack) trigger the induction of defense in plants prior to attack. I found that unwounded plants (*Brassica nigra*) that were regularly exposed to kairomones from snails (mucus and feces produced during movement of *Helix aspersa*) subsequently experienced reduced rates of attack by snails, unlike unwounded plants that received only one initial early exposure to snail kairomones. A follow-up experiment found that mucus alone did not affect snail feeding on previously harvested *B. oleracea* leaves, suggesting that changes in herbivory on *B. nigra* were due to changes in plant quality. The finding that chemicals associated with herbivores leads to changes in palatability of unwounded plants suggests that plants eavesdrop on components of non-volatile kairomones of their snail herbivores. Moreover, this work shows that the nature of plant exposure matters, supporting the conclusion that plants that have not been attacked or wounded nonetheless tailor their use of defenses based on incidental chemical information associated with herbivores and the timing with which cues of potential attack are encountered.

## Introduction

Animals often use information from their environment to gauge the risk of attack by predators [1], [2], [3]. This information may take the form of kairomones, chemical cues that are not directly associated with attack by a predator [1]. For animals, the value of information also often depends on the amount of information and when it is received: animals may give greater weight to cues that are received more recently or more often [4], [5]. Plants also utilize information regarding the risk of attack by herbivores: volatile cues from wounded neighbors [6], [7], [8], herbivore saliva [9], herbivore oviposition [10], and mechanical stimulation caused by moving herbivores [11] may all generate induced resistance against herbivores. Although kairomones are known to be important in animal systems [1], [2], [3] and are widespread in plant-microbe interactions [12], studies that evaluate kairomones in induction of plant defense are limited to a single recent study of volatile kairomones [13]. Similarly, just as animals may give greater weight to cues that are received more recently or more often [4], [5], plants might also be sensitive to the timing of cue detection and the amount of cue received, since the timing of kairomone cue presentation and the amount of cue received may be important indicators of the reliability of the information provided by the cue [14].

**Figure 1:**
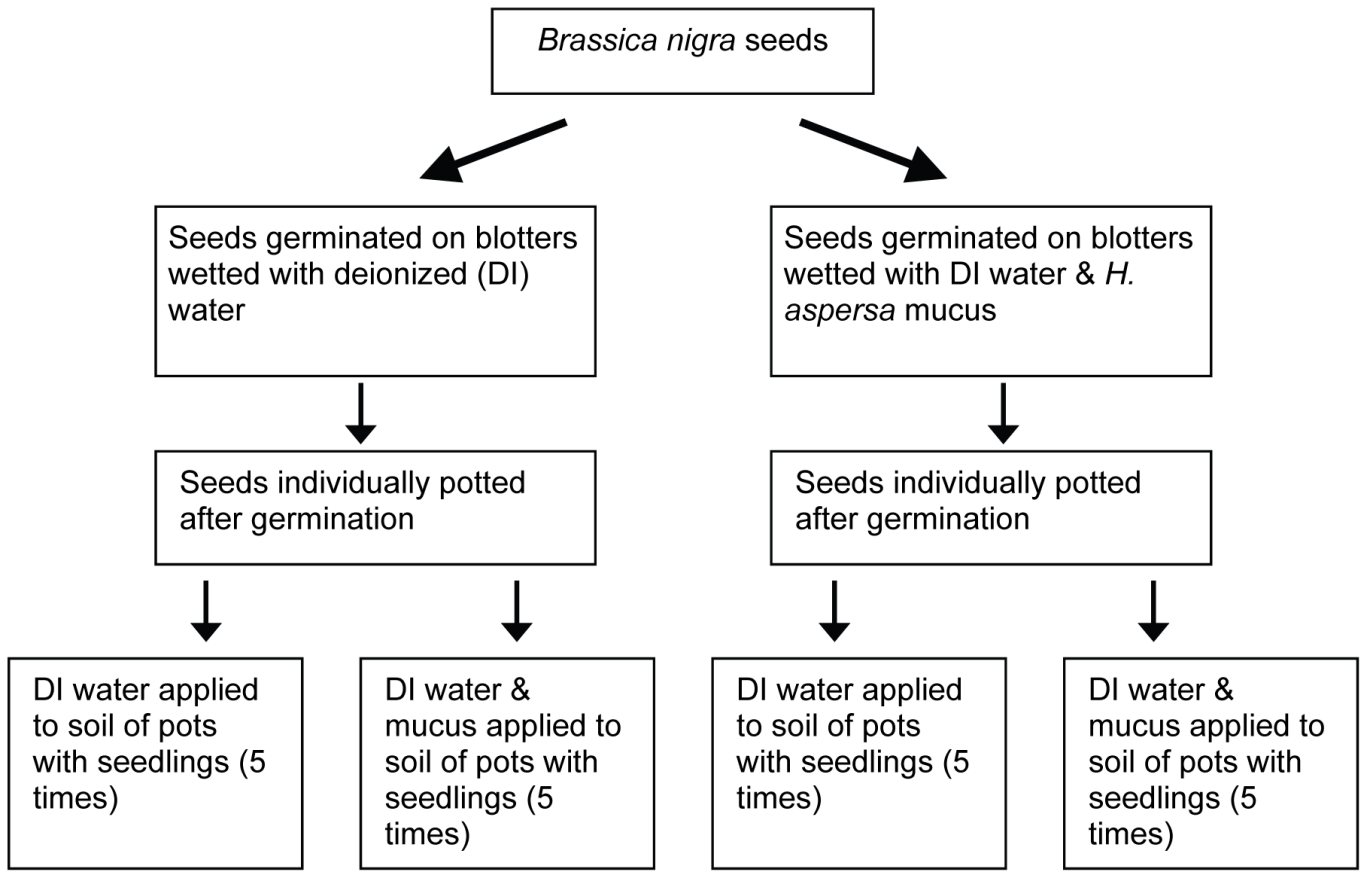
Diagrammatic description of the experimental design. The experimental design resulted in four treatment combinations of *Brassica nigra* plants depending upon the type of early cue and repeated cue the plants received: 1) plants that received deionized water (DI) once as a seed and received DI water five times as a seedling, 2) plants that received DI water as a seed and received DI water and mucus five times as a seedling, 3) plants that received DI water and mucus as a seed and only DI water five times as a seedling, 4) plants that received DI water and mucus as a seed and DI water and mucus five times as a seedling. In the diagram below, mucus refers to all substances present on blotters that were traversed by snails, such that mucus could also include snail feces (see text for full description).

In this paper, I describe a factorial experiment designed to determine whether 1) unwounded plants that are exposed to non-volatile kairomones of herbivores experience lower rates of herbivore attack and 2) whether the timing of kairomone exposure is important in determining changes in unwounded plant susceptibility to herbivore attack. I evaluated whether or not seedlings of a widespread annual plant species (*Brassica nigra*) experiences lower rates of herbivore attack after exposure to the non-volatile kairomone cue of a generalist herbivore, the common garden snail (*Helix aspersa*). Specifically, each plant was exposed to either snail mucus and deionized water or only deionized water to create one of four treatment combinations for each test plant (see Figure 1 for a diagram of the experimental design): exposure to mucus only as a seed (single early mucus exposure only), exposure to mucus five times while a seedling (repeated late mucus exposure only), exposure to mucus as a seed and a seedling (i.e. early and repeated mucus exposure), and no exposure as either a seed or a seedling). Mollusks are optimal herbivores for the study of non-volatile kairomone use by plants: snails and slugs are influential generalist herbivores in many terrestrial plant communities [15], [16], [17] capable of shaping the evolution of defense in *B. nigra* [18]. Defense against mollusk herbivory may be particularly important at the seedling stage, as seedlings are often more likely to be attacked by mollusks [19], [20], seedling age can be an important correlate of whether or not plants survive mollusk attack [21], and many of the community-level effects of mollusks arise because of death of plants at the seedling stage [16], [17], [22], [23]. Mollusks produce mucus for locomotion [15], a substance that provides plants with an incidental (yet likely reliable) cue of mollusk presence. Moreover, an enzyme in snail saliva leads to the induction of defense in marine algae [24], application of snail mucus to mechanically damaged plants triggers induction in *Arabidopsis thaliana* [25], and mollusk mucus may contain the plant hormone salicylic acid (J. Kästner, S. Meldau, et al., unpublished manuscript). All of this evidence suggest the potential for non-wounded plants to detect and utilize kairomones associated with snail mucus.

## Materials and Methods

### Study Species

*Brassica nigra* is an annual forb of Mediterranean origin that is now found in temperate grasslands worldwide. *Helix aspersa* is found in most of the world’s terrestrial plant communities and is known to consume a variety of plant species [26], [27]. Densities of *H*. *aspersa* can range widely in field settings. For example, in a four-year study near Santa Barbara, CA, [28] densities ranged from 0.55 snails/m^2^ to 24.76 snails/m^2^. *Brassica nigra* seeds were collected in 2006 from wild plants at the Sedgwick Reserve near Santa Ynez, CA. *Helix aspersa* were collected periodically from 2004–2007 in nearby Santa Barbara, CA. Snails were maintained in the lab at ambient temperature. Prior to the experiments, snails were maintained on a mixed diet of lettuce, plants from the Brassicaceae (e.g. kale, collards, mustards, and cabbage), and oatmeal.

### Experimental Exposure of Seeds and Seedlings to Snail Kairomones

This experiment was a 2×2 factorial design to investigate the effect of a single exposure to snail kairomones at the seed stage (i.e. seeds exposed to water or to water and and mucus) and the effect of a multiple exposures to snail kairomones at the seedling stage (i.e. seedlings experienced 5 exposures to water or to water and snail mucus; Figure 1). Mucus was collected from snails by placing 12 randomly selected adult snails overnight in each of four lidded translucent plastic containers (15.5 cm tall, 18 cm diameter); mean snail mass from 48 of the snails used in the trials was 7.43 g±0.37 SE. The bottom of each container was lined with eight 90 mm diameter germination blotter papers (Anchor Paper Co., St. Paul, MN) that were saturated with deionized water. Control (i.e. non-mucus) blotters were prepared in an identical way, except snails were not placed in the container with the blotters. There was no storage of collected mucus; blotters were used immediately after collection (e.g. blotters for use on April 6 were prepared on April 5).

On April 6, 2010, replicates were created by placing a single blotter in a 90 mm plastic Petri dish with 15 seeds of *B*. *nigra* placed atop the blotter. Seed placement was random on the blotter surface; mucus-treated blotters were covered with mucus and feces (mean proportion covered was 0.61±0.026 SE and 0.04±0.004 SE, respectively). Each treatment was replicated 32 times, for a total of 64 Petri dishes. Dishes were placed in a growth chamber with 14∶10 day:night photoperiod, 25 C:20 C temperature regime, and 60 percent relative humidity. Seeds were checked regularly (average time between checks was 9.31 hours ±1.92 SE) and deionized water was added as needed to keep blotters saturated. On April 7, seeds that had germinated in each treatment were randomly selected and singly planted into plastic greenhouse pots (5 cm×5.5 cm wide, 5.5 cm deep) filled with standard potting mix.

Planted seeds were kept in the same growth chamber and checked daily. Because plastic greenhouse pots were not connected, non-volatile water-soluble kairomones could not move among plants, whereas volatile components of snail kairomones, if present, were free to move among plants. All seedlings were watered with deionized water to maintain hydration. On April 13, 16, 19, 22, and 25, seedlings were watered using either deionized water or deionized water infused with snail mucus. The mucus/water treatments were prepared by adding approximately 400 ml of deionized water to the plastic containers with 8 mucus-coated blotters or plastic containers with mucus-free control blotters (these blotters set up in an identical manner to previous blotters) and thoroughly stirring the mixture. There was no difference in the mean amount of mucus covering blotters among any of the mucus exposure trials (F_5,18_ = 0.25, P = 0.93). During watering, care was taken to water directly into the soil to minimizing any contact with plant leaves. The treatment was applied to the soil and not also to the plant leaves because pre-emptive induction of defense would presumably be most advantageous if employed before the snail was in a position to attack the plant.

Herbivory trials were performed when plants were approximately 28 days of age (since germination date varied by approximately 24–30 hours). Twenty-five herbivory trials were conducted by placing a single adult snail and two randomly selected seedlings from the four factorial treatment groups into an arena constructed of two lidded translucent plastic containers (15.5 cm tall, 18 cm diameter). The first herbivory trial session occurred from 2 pm April 28, 2010 until 10 am April 29; the second session occurred from 12 pm April 29 until 8 am April 30, 2010. Leaf number, length, and width were recorded for all plants just prior to initiation of herbivory trials (total plant biomass is strongly correlated with leaf number, leaf length, and leaf width, unpublished data). After trials were concluded, the proportion of leaf tissue removed on each leaf and presence of snail visitation (i.e. presence of snail mucus) was visually estimated by a single observer.

### Additional Experiment to Determine Whether Mucus Alone Deters Feeding Snails

To evaluate whether or not mucus alone deterred snails and to determine whether visual estimates of herbivory were correlated with biomass loss, twenty blotters were placed in the bottom of plastic containers (9 cm diameter, 4.5 cm height) and wetted with reverse-osmosis water until saturated on June 24, 2013. Ten adult *H. aspersa* were placed overnight in individual dishes with an individual blotter, creating 10 mucus-treated blotters; 10 control blotters were created without *H. aspersa*. On June 25, 2013, one mucus and one control blotter were randomly selected and placed in the bottom of a plastic arena similar to the one used in the herbivory trials; this process was repeated for all blotters, resulting in 10 arenas. A 2×2 cm section of previously harvested *B. oleracea* leaf was weighed and placed on each blotter in each of the ten arenas (organic harvested *B. oleracea* was obtained from a local market). A single adult *H. aspersa* was added to each of the ten arenas. On June 26, the proportion of *B. oleracea* remaining was visually estimated, and all *B. oleracea* samples were washed to remove mucus, blotted dry, and weighed.

### Statistical Analyses

To evaluate whether there were differences in leaf number due prior to herbivory trials, a generalized linear model was used with a Poisson distribution. Leaf length prior to herbivory trials was evaluated using a general linear model. Following herbivory trials, a single linear mixed-effects model was used to evaluate the logit-transformed mean proportion of leaf area removed per plant [29]; snails were modeled as a random effect to accommodate variance among individuals selecting among pairs of plants. Fixed effects in the mixed model were the single early exposure treatment experienced by the plant as a seed (i.e. water or water and mucus), the repeated exposure treatment experienced by the plant as a seedling (water or water and mucus) and the interaction between the single early exposure treatment and the repeated exposure treatment. The logit transformation was applied to these data to meet assumptions for use in the linear model framework [29]; a small constant value of 0.01 was used during the transformation based upon examination of model residuals [29]. Use of this constant did not change the nature of the conclusions: similar qualitative outcomes were observed if I used non-transformed data or if the arcsine squareroot transformation was used.

## Results

Prior to herbivory trials, there was no difference in the total number of leaves on plants due to early exposure to mucus (Poisson generalized linear mixed model; z = −0.16, *P* = 0.88), repeated exposure to mucus (z = −0.24, *P* = 0.81), or their interaction (z = 0.52, *P* = 0.60). There was also no difference in the length of the longest leaf before herbivory trials as a function of early exposure to mucus (*F*_1,45_ = 0.15, *P* = 0.70), repeated exposure to mucus (*F*_1,45_ = 0.30, *P* = 0.59), or their interaction (*F*_1,45_<0.01, *P* = 0.93).

**Figure 2:**
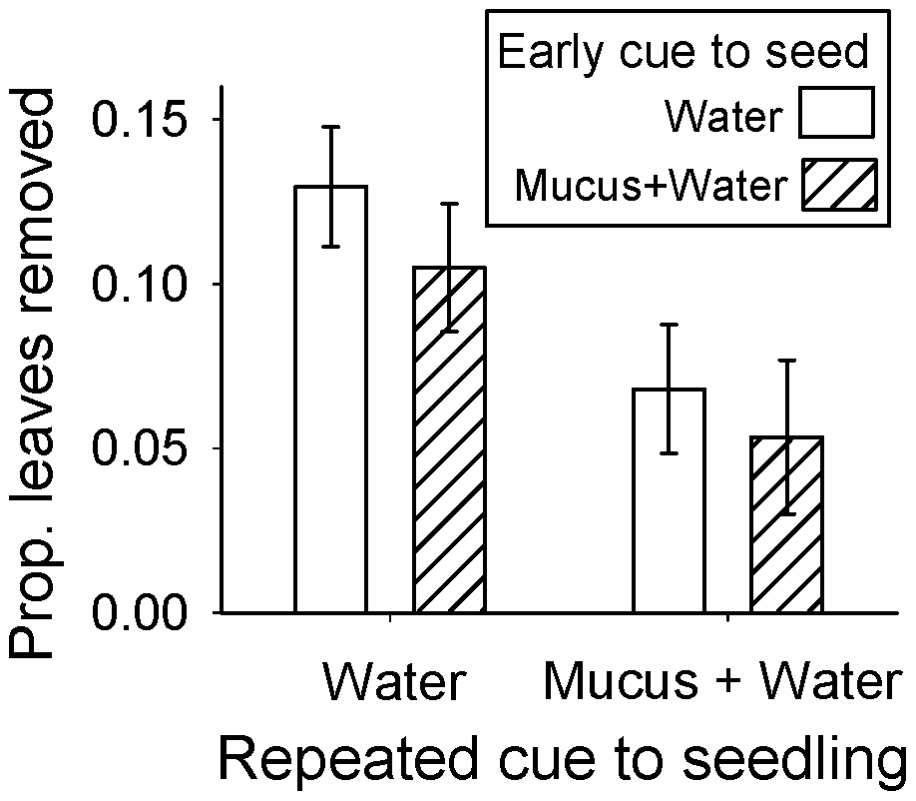
The timing and duration of exposure to herbivore kairomones (mucus from the snail *Helix aspersa*) determines the degree to which plants are attacked by snails. Early exposure of *Brassica nigra* seeds to snail mucus and deionized water did not lead to differences in herbivory by snails compared to seeds that received early exposure to only deionized water. However, plants that received repeated exposure to snail mucus and deionized water (five exposure events over the course of growth) experienced lowered rates of snail herbivory compared to seedlings that received repeated exposure to only water, regardless of early exposure as a seed. Data represent means ±1 S.E. of main effects for non-transformed data (statistical analyses in text use logit-transformed data).

Snail herbivory on seedlings was not significantly affected by early exposure to water versus snail mucus, although there was a trend of reduced herbivory in plants that experienced early exposure to snail mucus (*F*_1,22_ = 2.80, *P* = 0.109; Figure 2). In contrast, a large reduction in herbivory was observed for unwounded seedlings that received repeated exposure during the seedling stage: averaged across early exposure treatments, seedlings that received repeated mucus cue exposure experienced nearly half the herbivory experienced by water-treated controls (*F*_1,22_ = 11.68, *P* = 0.003; Figure 2). Plants that received both early and repeated exposure did not exhibit additional changes in palatability, i.e. there was no interaction between early exposure and repeated exposure (*F*_1,22_ = 0.71, *P* = 0.41) in affecting herbivory by snails.

Snail visitation, indicated by actual herbivory or by the presence of snail mucus on plants after herbivory trials, did not differ among treatment combinations (chi-square = 0.70, d.f. = 1, *P* = 0.80): snails visited 47 of the 50 test plants (94%). In the follow-up experiment, the presence of mucus alone was not a deterrent to snail feeding on *B. oleracea* (paired t-test, *t* = 1.40, d.f. = 9, *P* = 0.19); these results are expected to be conservative, since the mucus cues that would have been available to foraging snails in *B. nigra* herbivory trials would have represented a more dilute source of mucus and an older mucus cue. Visual estimation of the proportion of *B. oleracea* removed was significantly correlated with the change in *B. oleracea* biomass (*r* = 0.90, *t* = 8.64, d.f. = 18, *P*<0.001).

## Discussion

This work shows that a terrestrial plant species becomes less palatable to snail herbivores once exposed to non-volatile kairomone cues associated with snail herbivores and that the timing of cue presentation is critical for determining changes in plant palatability (Figure 2). Differences in rates of herbivory were not driven by snail preference for plants of a particular size or because snails avoided mucus cues per se, as there were no differences in the size or number of leaves among plants prior to herbivory trials, no differences in snail visitation to plants, and mucus itself did not deter snail feeding in choice trials with *B. oleoracea.* This work suggests that *B. nigra* utilized chemical information associated with herbivores to pre-emptively induce defenses prior to actual attack, and that repeated exposure to herbivore cues generated greater induction of plant defense. The non-volatile nature of mucus kairomones is confirmed by the significant differences in herbivory among seedlings that experienced repeated exposure to mucus (Figure 2), regardless of the treatments received by their neighbors that were less than 7 cm away (but rooted in a separate container). These findings have two main implications. First, they suggest that non-volatile kairomones are among the diverse types of information that plant utilize from their environment [13], [30], [31], [32], and such non-volatile kairomones may play roles in plant-herbivore interactions analogous to the roles they play in animal predator-prey interactions, i.e. the “ecology of fear”. Second, these findings illustrate that plants are sensitive to the timing of information, such that information timing changes the induction of defense in plants that have never experienced actual herbivory.

Much as kairomones have provided insight into the variation observed in animal populations [1], [3], this work suggests that non-volatile kairomone use may inform spatial and temporal variation observed in plant defense [33]. For example, recent work shows that plants respond to the volatile kairomones of specialist insect herbivores but not generalist insect herbivores [13]. The finding that plants respond to the water-borne mucus kairomones of generalist snail herbivores (Figure 2) but that they do not respond to volatile cues of generalist insect herbivores [13] suggests the interesting possibility that plants respond to non-volatile kairomones of generalist herbivores because mucus provides more accurate information about imminent attack compared to volatile cues of generalist arthropod herbivores. However, because timing of cue exposure is important (Figure 2) and timing of exposure differed between these studies, follow-up studies that utilize similar exposure protocols will be necessary to fully evaluate this hypothesis.

Much as temporal variation in information can mediate how animals respond to risk of potential predator attack [4], [5], [34], this work suggests that plants that have not been attacked by herbivores nonetheless give more weight to herbivore cues that are more frequent and more recently received. Such tailoring of investment is expected to be advantageous when information is reliable (i.e. snail mucus indicates risk of herbivory by snails) and when investment in defense comes at the expense of other components of plant fitness. Recent work has shown that plant defense, in the form of plants that fold their leaves in response to touch, is sensitive to the costs of defense: plants held their leaves closed for shorter periods of time when light resources were more limiting [31]. The work in this paper shows that induction of defense is also sensitive to the frequency with which plants receive chemical information (Figure 2) that may indicate a potential attack. Although these findings suggest that repeated exposure generates greater changes in plant palatability to snails, a strong trend of reduced palatability was also found for plants that only received a single early exposure to mucus (Figure 2), suggesting that early cue exposure may also be important.

In demonstrating that plants utilize non-volatile kairomones to induce defense, this work helps clarify several lines of future investigation for understanding the ecology of information use in plants. For example, this experiment exposed plants to complete mucus and all associated components, a scenario that reflects the information available to plants in natural settings, where snail mucus is provided with all associated components and mucus is not sterilized or otherwise altered. As a result, it is unknown whether plants in this study were responding to some inherent property of the mucus itself, or to organisms or components associated with snail mucus (e.g. mucus-borne bacteria, chemicals from consumed plants, or nutrients that are present in snail mucus); recent work suggests that mollusk mucus may contain the plant defense hormone salicylic acid (J. Kästner, S. Meldau, et al, unpublished manuscript). It is also possible that snail kairomones or associated components cause direct damage to plant roots or tissues, and this damage plays a role in induction. Although future studies are needed to evaluate the precise mechanism whereby chemicals associated with snails led to a change in the palatability of unwounded plants, the possibility snail mucus alone can lead to changes in plant defense is supported in both terrestrial and aquatic systems, as application of slug mucus to mechanically wounded areas of *A. thaliana* initiates the production of jasmonate [25], and the components of snail saliva cause induction of defense in marine algae [24]. Future studies will also be needed to determine the relative role of cue timing and cue amount: seedlings that received repeated mucus exposure in this study also received more total exposure to mucus and associated components. While this is likely to reflect plant-herbivore interactions in nature (e.g. plants in areas where snails are abundant likely receive mucus cues more often and in greater total amounts), studies that independently manipulate frequency and amount of exposure of plants to kairomone cues would help determine whether changes in cue quantity or timing alone are sufficient to generate changes in plant palatability to herbivores. Moreover, although this study shows that the susceptibility of unwounded plants to snails is affected by exposure to snail kairomones and that the nature of kairomone exposure is important, future studies that evaluate changes in plant gene expression and defensive chemistry will be essential for understanding the molecular basis of kairomone use by plants.
